# Bilateral Adrenal Masses, Extreme Catecholamine Excess, and Hypercalcitoninemia With Metastatic Neuroendocrine Neoplasia: A Diagnostic Dilemma

**DOI:** 10.7759/cureus.117409

**Published:** 2026-10-03

**Authors:** Ahmed Elhassan, Ahmed Mahgoub, Mohamed Abdel-Hadi Mohamed, Abu-Bekr Mohamed, Isra Ali

**Affiliations:** 1 Department of Endocrinology, Mohammed Bin Rashid University of Medicine and Health Sciences, Dubai, ARE; 2 Department of Internal Medicine, Tanta University Hospital, Tanta, EGY; 3 Faculty of Medicine, Omdurman Islamic University, Omdurman, SDN; 4 Department of Internal Medicine, Detroit Medical Center/Wayne State University School of Medicine, Detroit, USA; 5 Department of Biomedical Sciences, Mohammed Bin Rashid University of Medicine and Health Sciences, Dubai, ARE

**Keywords:** adrenal mass, calcitonin, catecholamines, men2b, metastatic disease, neuroendocrine tumor, pheochromocytoma

## Abstract

A 40-year-old man presented with back pain, urinary retention, mild unilateral weakness, weight loss, hypercalcemia, and newly recognized hypertension. During the early admission, blood pressure became markedly labile, reaching 225/106 mmHg. Contrast-enhanced computed tomography demonstrated approximately 10-cm bilateral heterogeneous adrenal masses and diffuse lytic skeletal metastases. Biochemical testing showed profound catecholamine excess, including urine metanephrine/creatinine 42,040 µg/g creatinine (reference range: 29-158 µg/g), normetanephrine/creatinine 6,079 µg/g creatinine (reference range: 53-659 µg/g), and vanillylmandelic acid/creatinine 212 mg/g creatinine (reference range: 1.1-4.1 mg/g). Serum calcitonin was 4,680 pg/mL (reference ≤8.4), with carcinoembryonic antigen (CEA) 239 ng/mL (reference <3.8 ng/mL for a non-smoker). Expert review of a bone biopsy showed a metastatic well-differentiated neuroendocrine tumor, WHO grade 3 (2017), with Ki-67 approximately 40%. The tumor expressed chromogranin, synaptophysin, pancytokeratin, thyroid transcription factor 1 (TTF-1), CEA, paired box 8 (PAX8), and cytokeratin (CK) 7 but was negative for calcitonin, thyroglobulin, CK20, GATA3, and S100. Small polypoid lesions at the tip of the tongue, considered possible mucosal neuromas, together with thyroid calcifications raised concern for multiple endocrine neoplasia type 2B; however, thyroid fine-needle aspiration, *RET* testing, and functional imaging were not completed before rapid clinical deterioration. The patient received alpha-blockade, palliative spinal radiotherapy, and carboplatin/etoposide; his course was complicated by profound pancytopenia, extended-spectrum beta-lactamase (ESBL)-producing *Escherichia coli* bacteremia, respiratory and renal failure, gastrointestinal bleeding, intracranial hemorrhage, and death. The case illustrates clinicopathologic discordance: the biochemical phenotype strongly favored pheochromocytoma, whereas metastatic-site histology did not establish the primary site.

## Introduction

Multiple endocrine neoplasia type 2B (MEN2B) is a rare autosomal dominant tumor-predisposition syndrome caused predominantly by activating germline variants in the *RET* proto-oncogene. It is characterized by medullary thyroid carcinoma (MTC), pheochromocytoma, and phenotypic findings that may include mucosal neuromas, intestinal ganglioneuromatosis, and a marfanoid habitus [1,2]. Pheochromocytomas in MEN2 are frequently bilateral or multifocal, whereas metastatic pheochromocytoma is uncommon [2,3].

The coexistence of bilateral adrenal masses, marked catecholamine excess, hypercalcitoninemia, and mucosal neuroma-like lesions strongly raises suspicion for MEN2B. Diagnostic interpretation becomes more difficult when metastatic tissue demonstrates neuroendocrine differentiation without unequivocally establishing its site of origin. Because pheochromocytoma and MTC may share neuroendocrine features, distinguishing them requires integration of tumor morphology and lineage-specific immunohistochemistry with the biochemical phenotype, imaging findings, and, when available, molecular testing [1,3,4].

We report the case of a 40-year-old man with severe labile hypertension, large bilateral adrenal masses, profound catecholamine excess, extreme hypercalcitoninemia, possible mucosal neuromas, and extensive lytic skeletal metastases with impending spinal cord compression. Expert review of a bone biopsy demonstrated metastatic well-differentiated neuroendocrine tumor, WHO grade 3, but did not establish the primary site. The discordance between the MEN2B-like clinicobiochemical phenotype and metastatic-site pathology, compounded by the inability to complete definitive molecular and functional investigations before rapid clinical deterioration, created an unusual diagnostic dilemma.

## Case presentation

A 40-year-old man without significant documented pre-existing comorbidity presented with back pain, urinary retention, mild unilateral weakness, and significant weight loss. At presentation, temperature was 36.4°C, heart rate 88 beats/minute, respiratory rate 18/min, oxygen saturation 100%, and blood pressure 158/104 mmHg. During the early admission, he developed severe and markedly labile hypertension, with recurrent readings above 180/110 mmHg and a documented peak of 225/106 mmHg.

Initial biochemical testing demonstrated hypercalcemia: serum calcium was 11.9 mg/dL and subsequently peaked at 12.5 mg/dL (reference range, 8.9-10.2 mg/dL). The initial parathyroid hormone (PTH) concentration was 15.1 pg/mL (reference range, 15-65 pg/mL), low-normal in the setting of hypercalcemia; 25-hydroxyvitamin D was 19.4 ng/mL (laboratory sufficiency range, 30-100 ng/mL). PTH later increased to 93 pg/mL after calcium had fallen to 9.0 mg/dL; therefore, the later value was not interpreted as evidence of primary hyperparathyroidism.

Contrast-enhanced computed tomography (CT) during the initial admission demonstrated large bilateral adrenal masses, approximately 10 cm each, with heterogeneous enhancement. The contemporaneous radiology interpretation favored bilateral adrenal metastases. Diffuse lytic lesions involved the visualized axial and appendicular skeleton, consistent with extensive osseous metastatic disease. The lungs were clear with no mediastinal or hilar lymphadenopathy; the liver, spleen, pancreas, and kidneys were reported as unremarkable, and no sizeable para-aortic lymphadenopathy was identified (Figure 1).

**Figure 1:**
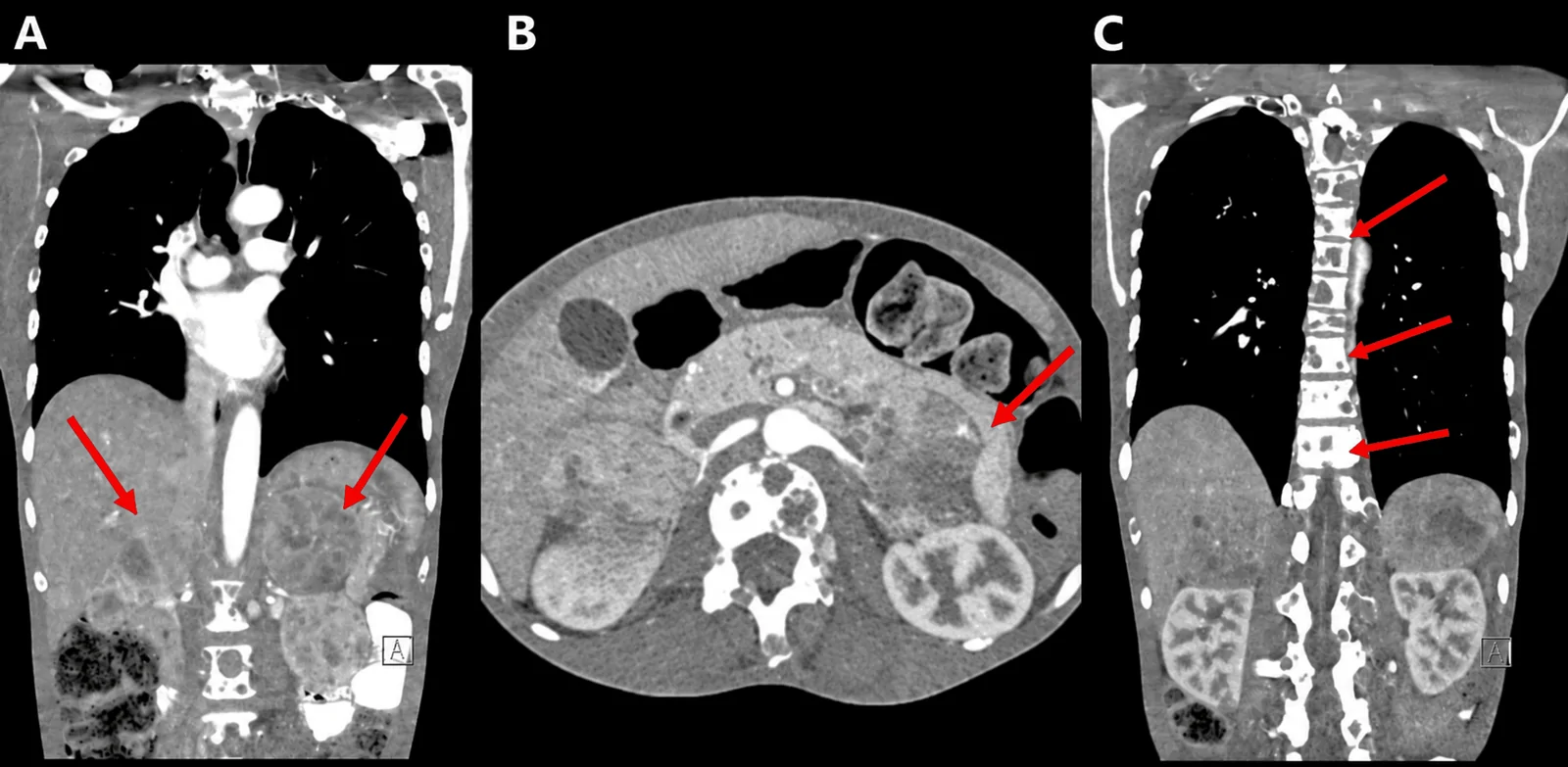
Contrast-enhanced CT demonstrating bilateral adrenal masses and extensive skeletal metastatic disease (A) Coronal image demonstrating bilateral adrenal masses (arrows). (B) Axial image demonstrating the left adrenal mass (arrow). (C) Coronal image demonstrating multiple lytic vertebral lesions (arrows).

Spinal magnetic resonance imaging (MRI) demonstrated extensive metastatic disease with vertebral involvement. Brain MRI additionally demonstrated an abnormality in the left pterygoid region. The formal radiology report described T1/T2 hyperintense signal with hyperostosis of the left pterygoid body, recommended CT/bone-scan correlation, and identified no intracranial mass lesion (Figure 2).

**Figure 2:**
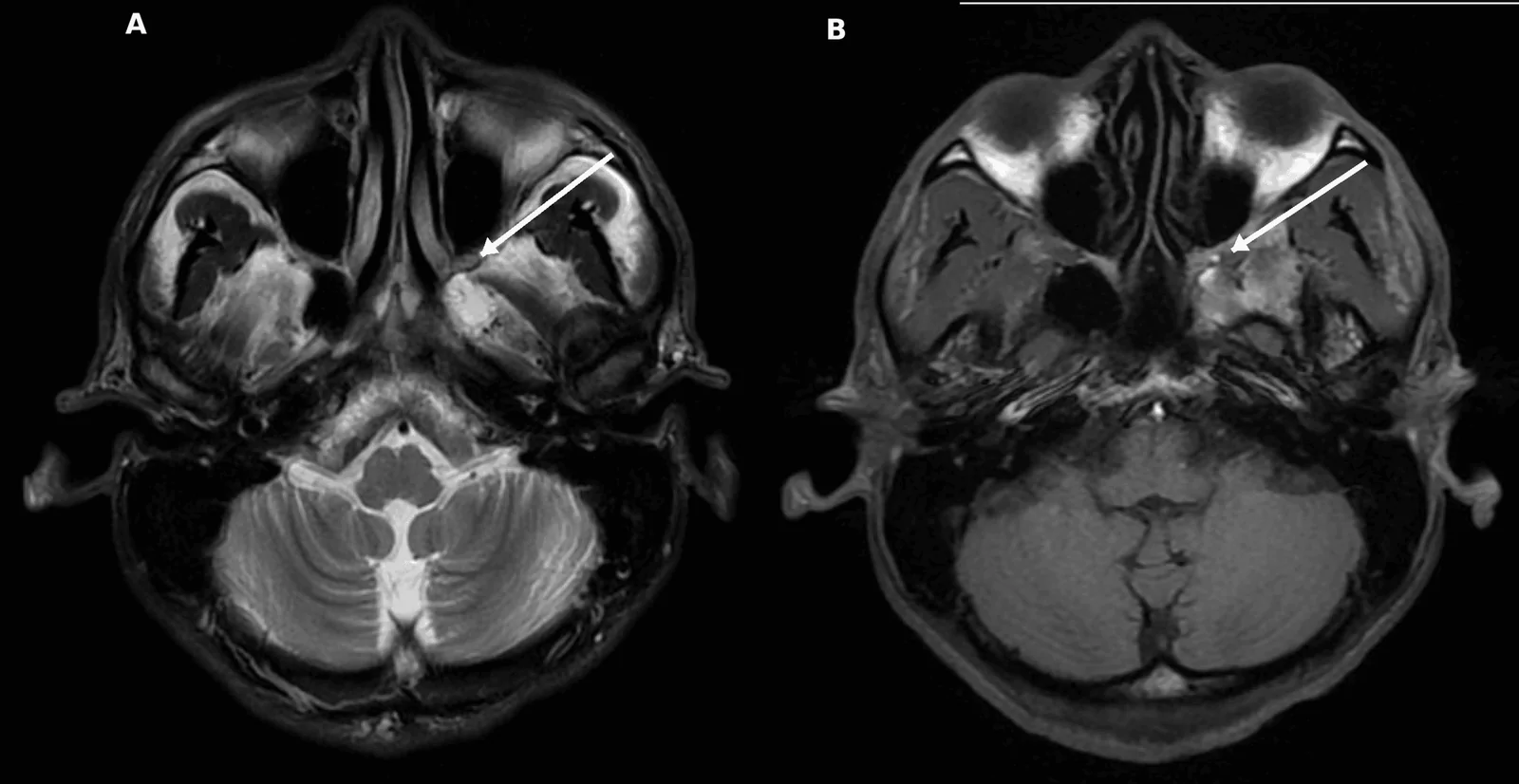
Brain MRI (axial view) demonstrating the left pterygoid-region abnormality T1/T2 hyperintense signal with hyperostosis of the left pterygoid body (arrows). No intracranial mass lesion was identified.

Endocrine evaluation demonstrated profound catecholamine excess (Table 1). Urine metanephrine/creatinine was 42,040 µg/g creatinine (reference range, 29-158 µg/g), approximately 266 times the upper reference limit, with marked elevations in normetanephrine and catecholamines. Plasma adrenaline and noradrenaline were also markedly elevated. Together with the bilateral adrenal masses and severe labile hypertension, these findings made catecholamine-secreting adrenal disease, particularly pheochromocytoma, the leading clinical diagnosis.

**Table 1:** Key biochemical and hormonal findings during admission PTH, parathyroid hormone; VMA, vanillylmandelic acid; ACTH, adrenocorticotropic hormone; 25-OH vitamin D, 25-hydroxyvitamin D; CEA, carcinoembryonic antigen; CA 19-9, carbohydrate antigen 19-9

Test	Patient Value	Reference Range
Calcium	11.9 mg/dL initially; peak 12.5 mg/dL	8.9-10.2 mg/dL
PTH	15.1 pg/mL	15-65 pg/mL
Urine metanephrine/creatinine	42,040 µg/g creatinine	29-158 µg/g creatinine
Urine normetanephrine/creatinine	6,079 µg/g creatinine	53-659 µg/g creatinine
Urine adrenaline/creatinine	1,530 µg/g creatinine	<23 µg/g creatinine
Urine noradrenaline/creatinine	757 µg/g creatinine	<114 µg/g creatinine
Urine dopamine/creatinine	360 µg/g creatinine	<422 µg/g creatinine
VMA/creatinine	212 mg/g creatinine	1.1-4.1 mg/g creatinine
Plasma adrenaline	>2,560 ng/L	≤82 ng/L
Plasma noradrenaline	>14,500 ng/L	≤499 ng/L
24-h urinary free cortisol	2,660 µg/24 h	21-292 µg/24 h
Calcitonin	4,680 pg/mL	≤8.4 pg/mL
CEA	239 ng/mL	<3.8 ng/mL
CA 19-9	150 U/mL	<39 U/mL
Morning cortisol	837 nmol/L; repeat 780 nmol/L	133-537 nmol/L
ACTH	27.5 and 40.9 pg/mL	<46.0 pg/mL
25-OH vitamin D	19.4 ng/mL	Sufficiency: 30-100 ng/mL

A 24-hour urine collection demonstrated urinary free cortisol of 2,660 µg/24 hours (reference range, 21-292 µg/24 hours), approximately 9.1 times the upper reference limit. Morning cortisol was 837 nmol/L and remained elevated at 780 nmol/L (reference range, 133-537 nmol/L). Adrenocorticotropic hormone (ACTH) values were 27.5 and 40.9 pg/mL (reference, <46.0 pg/mL). These results documented marked hypercortisolism, but acute severe illness and incomplete confirmatory testing prevented assignment of a specific endogenous Cushing syndrome diagnosis [5].

Serum calcitonin was 4,680 pg/mL (reference ≤8.4 pg/mL), more than 550 times the upper reference limit. Carcinoembryonic antigen (CEA) was 239 ng/mL (reference <3.8 ng/mL for a non-smoker), and carbohydrate antigen (CA) 19-9 was 150 U/mL (reference <39 U/mL). Clinical documentation later described small polypoid lesions at the tip of the tongue, raising the possibility of mucosal neuromas. Thyroid ultrasonography later during the admission showed bilateral thyroid enlargement with heterogeneous echotexture and multiple calcifications involving both lobes (Figure 3). MEN2B with medullary thyroid carcinoma (MTC) and pheochromocytoma was therefore considered clinically. Thyroid fine-needle aspiration and *RET* germline testing were not completed before the patient's rapid clinical deterioration.

**Figure 3:**
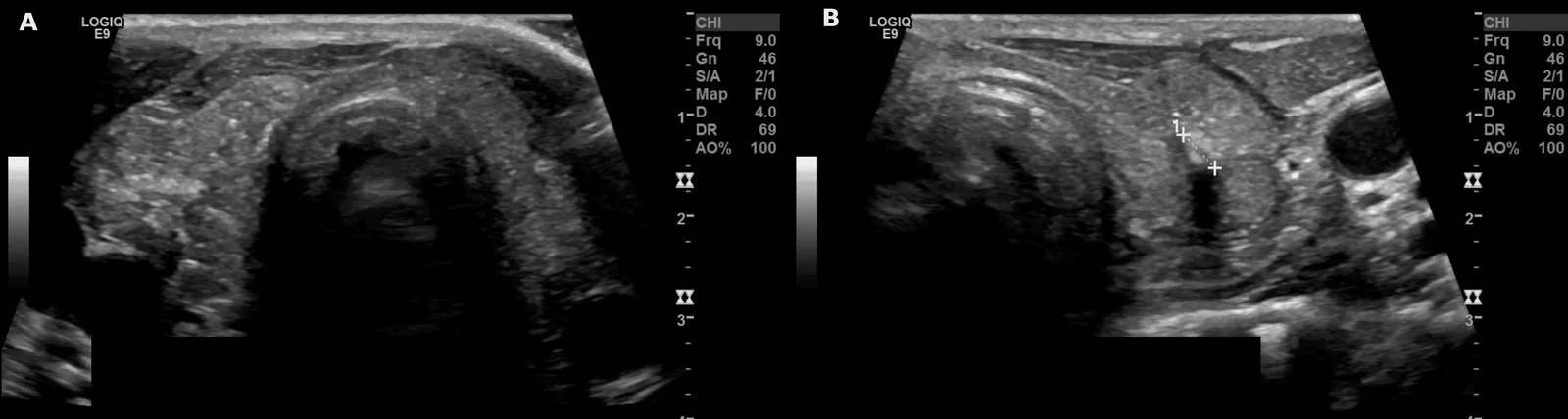
Thyroid ultrasonography (transverse view) demonstrating heterogeneous thyroid echotexture with multiple bilateral calcifications The formal ultrasound report described bilateral thyroid enlargement, with reported lobe volumes of 7.1 mL on the right and 6.9 mL on the left.

Alpha-adrenergic blockade with prazosin was initiated at 1 mg twice daily and progressively titrated, reaching up to 8 mg three times daily before subsequent adjustment. Additional blood-pressure and heart-rate control included labetalol, amlodipine, and later bisoprolol, with intravenous volume expansion.

The bone biopsy subsequently underwent external expert review a month after presentation (Table 2). The tumor showed organoid architecture with small nests and trabeculae of round-to-spindle cells. Up to five mitoses per 10 high-power fields were identified without necrosis. Ki-67 exceeded 20% and was estimated at approximately 40%. The final expert diagnosis was metastatic well-differentiated neuroendocrine tumor, WHO grade 3 (2017 classification); small-cell and large-cell neuroendocrine carcinoma were excluded. The 2017 WHO classification introduced the well-differentiated grade 3 neuroendocrine tumor category, separating it from poorly differentiated neuroendocrine carcinoma [6].

**Table 2:** Expert pathology review WHO, World Health Organization; HPF, high-power field; NEC, neuroendocrine carcinoma; AE1/AE3, pancytokeratin AE1/AE3; TTF-1, thyroid transcription factor 1; CEA, carcinoembryonic antigen; PAX8, paired box 8; CK, cytokeratin; CDX2, caudal-type homeobox 2; GATA3, GATA-binding protein 3; PSA, prostate-specific antigen; LCA, leukocyte common antigen.

Feature	Finding
Diagnosis	Metastatic well-differentiated neuroendocrine tumor, WHO grade 3 (2017)
Mitoses	Up to 5/10 HPF
Necrosis	Absent
Ki-67	Approximately 40%
Positive	Chromogranin, synaptophysin, AE1/AE3, TTF-1, CEA, PAX8, CK7
Negative	Calcitonin, thyroglobulin, CK20, CDX2, GATA3, PSA, desmin, S100, Melan-A, CD117, DOG1, CD30, LCA
p53	Weak/focal, <5%
Interpretation	Primary site unresolved; calcitonin-negative MTC considered; small- and large-cell NEC excluded

Immunohistochemistry demonstrated positivity for chromogranin, synaptophysin, pancytokeratin AE1/AE3, TTF-1, CEA, PAX8, and CK7, with CK20 negativity. Calcitonin, thyroglobulin, CDX2, GATA3, PSA, desmin, S100, Melan-A, CD117, DOG1, CD30, and LCA were negative. p53 staining was weak/focal in fewer than 5% of tumor cells. The expert review considered several potential primary sites, including calcitonin-negative MTC, but did not establish the primary site. The epithelial marker expression and GATA3 negativity were atypical for conventional pheochromocytoma/paraganglioma (PPGL) despite the compelling clinicobiochemical phenotype [1].

Given extensive skeletal disease and impending spinal cord compression, high-dose corticosteroids and palliative spinal radiotherapy were administered. Carboplatin plus etoposide was initiated during the admission on the basis of the contemporaneous clinical assessment and preliminary pathologic diagnosis of an aggressive metastatic neuroendocrine neoplasm. Following the first cycle, the patient developed profound pancytopenia complicated by neutropenic sepsis with extended-spectrum beta-lactamase (ESBL)-producing *Escherichia coli* bacteremia. He subsequently developed respiratory failure, gastrointestinal bleeding, and renal failure requiring intensive supportive treatment. Despite treatment, he deteriorated, developed an intracranial hemorrhage, and died during the same hospitalization.

## Discussion

This case presents an unusually difficult intersection between the clinicobiochemical phenotype of metastatic pheochromocytoma and findings suggestive of MEN2B/MTC. The bilateral adrenal masses measuring approximately 10 cm, profound metanephrine and catecholamine excess, and severe labile hypertension strongly favored catecholamine-secreting adrenal tumors. Contemporary WHO classification emphasizes integration of morphology with functional and lineage-specific biomarkers; conventional PPGL diagnosis requires integration of morphology, immunophenotype, biochemical findings, and clinical context [1]. However, the metastatic biopsy was chromogranin/synaptophysin positive but also pancytokeratin positive and GATA3 negative, creating a central clinicopathological discordance: the biochemical and radiologic phenotype strongly favored catecholamine-secreting adrenal disease, whereas the metastatic-site immunophenotype did not establish PPGL origin [1].

The second major diagnostic signal was extreme hypercalcitoninemia. MEN2B combines MTC and pheochromocytoma and may feature mucosal neuromas [2]. Jester et al. reported a closely comparable case in which a patient with MEN2B presented with diffuse osseous metastatic disease, an adrenal lesion, elevated calcitonin and CEA, and markedly elevated metanephrines [4]. In their case, bone biopsy favored metastatic pheochromocytoma because calcitonin, PAX8, TTF-1, and keratin stains were negative; MTC was separately confirmed by thyroid fine-needle aspiration and *RET* p.M918T was demonstrated. Our patient shared much of the phenotype but lacked the decisive thyroid pathology, *RET* result, and functional imaging, while the metastatic immunophenotype was substantially different.

Metastatic pheochromocytoma is uncommon in MEN2. A systematic review of cluster 2-related metastatic PPGL identified 29 MEN2 cases; bilateral adrenal tumors were frequent, and median primary tumor size in MEN2 metastatic pheochromocytoma approached 10 cm [3]. Distinguishing pheochromocytoma metastases from MTC metastases is difficult because both are neuroendocrine and may express chromogranin and synaptophysin. Previous MEN2A cases demonstrate the usefulness of metastatic-site calcitonin staining and postoperative biochemical behavior in determining lineage [3,7].

Although calcitonin 4,680 pg/mL made occult MTC a major concern, hypercalcitoninemia is not absolutely specific for MTC. Rare pheochromocytomas have been associated with calcitonin elevation; normalization after adrenalectomy without MTC has been reported, and direct ectopic calcitonin secretion by pheochromocytoma has been demonstrated [8,9]. Thus, coexisting occult MTC and pheochromocytoma in an MEN2B-like phenotype and calcitonin production associated with the adrenal/neuroendocrine tumor both remained biologically plausible.

The initial low-normal PTH in the presence of hypercalcemia argued against straightforward MEN2-associated primary hyperparathyroidism as the cause of the presenting hypercalcemia. Likewise, urinary and serum cortisol concentrations were markedly elevated, but severe acute illness and incomplete confirmatory testing prevented a reliable diagnosis of endogenous Cushing syndrome; accepted diagnostic guidance recommends confirmation with appropriate additional testing before etiologic classification [5].

In the current case, the expert pathology review classified the metastasis as a well-differentiated grade 3 neuroendocrine tumor, with Ki-67 approximately 40%, limited mitotic activity, and no necrosis, while explicitly excluding small-cell and large-cell neuroendocrine carcinoma. This distinction is consistent with the 2017 WHO framework separating well-differentiated grade 3 neuroendocrine tumors from poorly differentiated neuroendocrine carcinomas [6]. This expert opinion should supersede preliminary descriptions in the clinical record. Nevertheless, it did not establish the organ of origin.

The rapid clinical course prevented the investigations most likely to resolve the dilemma: *RET* germline testing, thyroid tissue diagnosis, and PPGL-directed or somatostatin-receptor functional imaging. Contemporary classification and disease-specific guidance emphasize integration of morphology, immunophenotype, biochemical findings, imaging, and molecular testing when tumor lineage is uncertain [1,2,6]. When pathology, biochemical phenotype, and imaging do not converge, diagnostic uncertainty should be preserved rather than retrospectively resolved by assumption.

## Conclusions

This case demonstrates the diagnostic challenge posed by metastatic neuroendocrine disease when biochemical, radiologic, and pathologic findings point toward overlapping endocrine tumor syndromes. Bilateral large adrenal masses, extreme catecholamine excess, and severe labile hypertension made pheochromocytoma highly compelling clinically, while profound hypercalcitoninemia and possible mucosal neuromas raised concern for MEN2B and occult MTC. However, the metastatic tumor's epithelial immunophenotype and negative calcitonin staining prevented definitive assignment to either lineage.

The most defensible final interpretation is metastatic well-differentiated grade 3 neuroendocrine neoplasia of uncertain primary origin occurring in a patient with a highly suggestive catecholamine-secreting bilateral adrenal phenotype and clinical features suspicious for MEN2B. Definitive syndromic and tumor-lineage classification was precluded by rapid deterioration before *RET* testing, thyroid tissue confirmation, and functional imaging could be completed. Preserving this uncertainty is central to the teaching value of the case.
